# Suboptimal Plasma Long Chain *n*-3 Concentrations are Common among Adults in the United States, NHANES 2003–2004

**DOI:** 10.3390/nu7125534

**Published:** 2015-12-09

**Authors:** Rachel A. Murphy, Elaine A. Yu, Eric D. Ciappio, Saurabh Mehta, Michael I. McBurney

**Affiliations:** 1 Nutrition Science and Advocacy, DSM Nutritional Products, 45 Waterview Blvd, Parsippany, NJ 07054, USA; eric.ciappio@dsm.com (E.D.C.); michael.mcburney@dsm.com (M.I.M.); 2 School of Population and Public Health, University of British Columbia, 2206 East Mall, Rm 167, Vancouver, BC V6T 1Z3, Canada; 3 Division of Nutritional Sciences, Cornell University, 314 Savage Hall, Ithaca, NY 14853, USA; eay27@cornell.edu (E.A.Y.); smehta@cornell.edu (S.M.)

**Keywords:** omega-3, polyunsaturated fatty acids, NHANES, adequacy, dietary intake

## Abstract

Population data on long-chain omega-3 polyunsaturated fatty acid (LCn-3 PUFA) status from biomarkers of dietary intake is lacking. The objectives were to describe plasma LCn-3 PUFA concentrations and compare them to concentrations associated with cardiovascular health and dietary recommendations for two servings of seafood/week. Fasting plasma fatty acids were measured among 1386 subjects ≥20 years from the National Health and Nutrition Examination Survey, 2003–2004. LCn-3 concentrations represent the sum of eicosapentaenoic acid, docosapentaenoic acid and docosahexaenoic acid relative to total fatty acids (expressed as a percentage). Mean LCn-3 PUFA concentration was 2.07% (95% CI 1.95–2.19). Overall, 80.6% of participants had LCn-3 below concentrations recommended for cardiovascular health. Hispanic participants were the most likely to have LCn-3 PUFA below recommended levels. Nearly all participants (95.7%) had LCn-3 below concentrations associated with cardiovascular protection. Older participants (≥60 years) had higher LCn-3 PUFA concentrations than those aged 20–39 years but not aged 40–59 years. LCn-3 PUFA concentrations were lower for Hispanic participants relative to non-Hispanic black participants. Suboptimal LCn-3 concentrations are common among U.S. adults. These findings highlight the need to increase LCn-3 intake among Americans.

## 1. Introduction

In 2010, the Dietary Guidelines Advisory Committee (DGAC) recommended seafood intake be increased to two servings (eight ounces) per week for adults as part of a healthy eating pattern [1]. The recommendation stems from clinical [2] and observational studies [3,4] that reported cardiovascular benefits of at least eight ounces or approximately 250 mg per day of long-chain (LC) *n*-3 polyunsaturated fatty acids (PUFA). The American Heart Association also recommends at least 2 servings of fish per week, especially oily fish rich in LCn-3 PUFA; eicosapentaenoic acid (EPA) and docosahexaenoic acid (DHA) [5]. However, dietary intake of seafood is consistently low across all ages for men and women in the United States [6]. Less than 10% of the population met the 2010 DGAC recommendation [7] prompting the 2015 DGAC to once again highlight the need to meet intake recommendations for seafood.

Most of the data on seafood and LCn-3 PUFA intake in large populations were derived from food frequency questionnaires, 24-h recall/record or multi-day recalls/records. Self-reported dietary data may introduce bias due to the difficulty obtaining precise and accurate information [8]. Circulating concentrations of LCn-3 PUFA provide a biomarker of short-term dietary fatty acid intake and are a more accurate characterization of LCn-3 status as well as associated health risks. Two cohort studies in the U.S. reported mean dietary intake of EPA and DHA below recommendations and correspondingly low plasma or serum LCn-3 PUFA [9,10], however, neither of these studies were in populations representative of the U.S.

Between 2003 and 2004 the National Health and Nutrition Examination survey (NHANES) measured plasma fatty acids of adults in the U.S. This provides a unique opportunity to evaluate the LCn-3 PUFA status in a nationally representative sample. The Centers for Disease Control and Prevention (CDC) published a descriptive summary of the absolute amounts of individual fatty acids [11] within NHANES, but more detailed interpretation is needed given the emphasis on dietary intake and well-established cardio-protective benefits of LCn-3 PUFA. Data on LCn-3 concentrations (relative percent of total fatty acids) is also important to facilitate comparison with other studies that predominately report fatty acids as concentrations [12]. Thus, our objective was to examine plasma LCn-3 PUFA concentration in NHANES and to compare concentrations with plasma and serum levels of LCn-3 PUFA from prior studies that correspond to dietary recommendations of >250 mg EPA and DHA per day and cardiovascular health.

## 2. Materials and Methods

This cross-sectional analysis included nationally representative data from NHANES (2003–2004), conducted by the National Center for Health Statistics (NCHS) at the CDC. Details on the complex survey design and multistage probability sampling of non-institutionalized U.S. civilians have been previously documented [13]. We excluded participants likely to differ in fatty acid requirements due to demands for growth and development; pregnant or breastfeeding women, and participants aged <20 years. We further excluded participants missing data on any individual fatty acids as that would affect calculation of total fatty acids. The resulting subsample was 1386 participants.

Details of data collection on the blood samples and interviews can be found in NHANES documentation [11]. Briefly, blood was drawn from adults ≥20 years after fasting for ≥8 h. Plasma samples were stored at −70 °C until measurement in 2010–2011. Gas chromatography-mass spectrometry was used to measure 24 plasma fatty acids using a modification of the method of Lagerstedt *et al.* [14]. The concentrations of fatty acids were expressed as a relative percent of the sum of the 24 fatty acids reported in NHANES as shown in Table S1. The concentration of LCn-3 PUFAs was determined in the same manner as previous publications [9,15,16], as the sum of EPA, DHA and docosapentaenoic acid (DPA) divided by total fatty acids (See Table S1 for all fatty acids included in the “total fatty acids” denominator).

### 2.1. Comparison with Other Studies

To provide context to fatty acids levels we compared plasma LCn-3 in NHANES to published epidemiologic studies. Although self-reported measures of diet introduce bias, dietary recommendations are based around achieving an intake amount not biochemical levels. Thus, to provide a dietary reference for plasma levels in NHANES, we used data from the Nurses’ Healthy Study [9]. Sun *et al.* [9] reported moderate-to-strong correlations between dietary LCn-3 assessed by food frequency questionnaire, and plasma LCn-3. The mean plasma LCn-3 concentration was 2.49% and mean daily intake of EPA, DPA and DHA was 260 mg (calculated from mean dietary LCn-3 of 2.52% of total fat intake multiplied by mean total dietary fat of 63.6 g/day), which approximates the 2010 DGAC recommendation for intake [1]. We defined “optimal” status of plasma LCn-3 as a level that may convey health benefits. The cutpoint for optimal was based on the results of Virtanen *et al.* [17], who observed a 53% and 46% lower risk of sudden cardiac death among subjects in the tertile 2 and tertile 3 of serum LCn-3 fatty acids. We used a conservative approach to compare NHANES LCn-3 concentrations with those associated with risk in Virtanen *et al.* [17] by selecting the lowest limit of tertile 2 which ranged from 3.86% to 4.95%. The matrix that fatty acids are measured in (e.g., red blood cells, plasma phospholipids or whole blood) are strongly correlated; *r* = 0.91 [18] and previous studies [12,18] have used a similar approach to facilitate comparison to other studies or combine measures of fatty acids from different studies.

### 2.2. Statistical Analysis

Results are reported for the total analytical population and stratified by age, and race, which were determined *a priori*. Age was categorized into three age groups of roughly 20 years spans that approximate life stages; younger adult, middle age and older age. Race was self-identified and based on the 2000 U.S. Census race and ethnicity categories (non-Hispanic white, non-Hispanic black, Mexican American, other Hispanic and other, where all else was labeled other including mixed race). Race is presented separately for non-Hispanic white, non-Hispanic black and Hispanic, while “other” is included in the “all” group and not separately due to limited sample size (*N* = 60).

Sample sizes are presented as unweighted. All other data is weighted by the 2-year fasting weights specific to the subsample with fatty acid measures (WTSAF2YR). The 2-year fasting weights accounts for the additional probability of selection into the subsample component that provided fasting blood samples, as well as additional nonresponse. Means and standard error (SE) or 95% confidence intervals (CI) are reported for continuous variables. The prevalence (%) of inadequate LCn-3 below recommended levels is reported for all participants and by age and race. Differences between groups were determined by linear regression accounting for study weight. Significance was set at a Bonferroni adjusted α of *p* < 0.003125 (*p* < 0.05 divided by 16 (4 race groups and 4 age groups)). All statistical analyses were conducted with Stata version 13 (StataCorp LP, College Station, TX, U.S.).

## 3. Results

Participant characteristics are shown in Table 1 for all participants (Column 1) and by race (Columns 2 through 4). Characteristics are shown for descriptive purposes and were not statistically compared. The overall population was approximately equal men and women. The majority of participants had at least high school education with the exception of Hispanics. The mean BMI was overweight or obese (non-Hispanic black participants). Less than one-third reported current smoking and all races, less than 10% had prevalent cardiovascular disease (CVD).

In a linear regression model age (*p* < 0.001) and race (*p* = 0.004) were significantly associated with LCn-3 PUFA but gender was not (*p* = 0.74), and thus data are shown for men and women together. The overall mean LCn-3 PUFA was 2.07% (95% CI 1.95–2.19). The mean LCn-3 PUFA was 2.05% (95% CI: 1.91–2.19) for men and 2.10% (95% CI: 1.98–2.21) for women aged ≥20 years. Table 2 shows the mean of LCn-3 PUFA concentrations for the study population by race and age. Older participants (≥60 years) had higher LCn-3 PUFA than those aged 20–39 years (*p* = 0.001) but not aged 40–59 years (*p* = 0.01). LCn-3 PUFA was similar between participants aged 20–39 and 40–59 years (*p* = 0.46). LCn-3 PUFA concentrations were similar for Hispanic participants aged 20 and older relative to non-Hispanic white participants (*p* = 0.04) and among non-Hispanic black participants relative to non-Hispanic white (*p* = 0.02). LCn-3 PUFA was lower among non-Hispanic black participants (*p* < 0.0001). Additional percentiles of LCn-3 PUFA can be found in Table S2.

Across all ages, approximately 8 in 10 participants (80.6%) had plasma LCn-3 PUFA concentrations below what is associated with dietary intake levels recommended by the 2010 and 2015 DGAC and the American Heart Association (Table 3). The prevalence of LCn-3 PUFA below recommended levels ranged from a high of 94.5% among Hispanic participants aged 20–39 years to 53.6% of non-Hispanic black participants ≥60 years. Hispanic participants were the most likely to have LCn-3 PUFA below recommended levels (*p* = 0.0017). Nearly all (95.7%) participants had LCn-3 PUFA below concentrations associated with cardio-protection. This was particularly evident among Hispanic participants of any age, as <2% had LCn-3PUFA above levels associated with cardio-protection, although the difference was not significant (*p* = 0.0077) at the Bonferroni adjusted α level of 0.00325.

**Table 1:** Characteristics of study participants, *N* = 1386.

	All	Hispanic	Non-Hispanic White	Non-Hispanic Black
Women, *N* (%)	693 (48.5)	158 (43.4)	374 (52.2)	130 (55.2)
Age, *N* (%)				
≥20 years	1386	334 (11.3)	739 (72.1)	253 (10.9)
20–39 years	412 (37.0)	101 (55.1)	196 (33.0)	99 (48.3)
40–59 years	423 (40.1)	96 (32.2)	223 (41.6)	78 (33.0)
≥60 years	551 (23.0)	137 (12.7)	320 (25.5)	76 (18.7)
Education, *N* (%)				
<High school	411 (18.4)	202 (51.2)	116 (11.4)	80 (30.0)
High school	339 (25.6)	65 (23.3)	203 (26.7)	59 (24.2)
>High school	634 (55.9)	67 (25.5)	420 (61.9)	112 (45.6)
BMI, kg/m^2^, Mean ± SE	28.3 ± 0.20	29.0 ± 0.38	28.0 ± 0.25	30.2 ± 0.52
Smoking status, *N* (%)				
Never	687 (49.9)	177 (52.7)	332 (47.7)	145 (60.0)
Current	307 (25.0)	57 (21.4)	168 (25.3)	69 (27.7)
Former	392 (25.1)	100 (25.9)	69 (27.7)	39 (12.3)
Prevalent CVD, *N* (%)	159 (8.08)	27 (3.70)	99 (8.41)	22 (6.84)

BMI: body mass index. “All” includes individuals who self-identified with a race/ethnicity other than Hispanic, non-Hispanic white and non-Hispanic black. Hispanic includes Mexican Americans and other Hispanic. Data presented as *N* (weighted column percent) except for age ≥20 years which is presented as *N* (weighted row percent) or weighted mean (SE) for continuous variables. Prevalent cardiovascular disease (CVD) defined as self-report congestive heart failure, coronary heart disease, angina, angina pectoris, heart attack or stroke.

**Table 2:** Mean long chain *n*-3 PUFA status (% of total fatty acids: EPA + DPA + DHA) in all participants and by race and age.

	All	Hispanic	Non-Hispanic White	Non-Hispanic Black
**≥20 years**				
Mean (95% CI)	2.07 (1.95–2.19)	1.82 (1.65–2.00) ^a^	2.04 (1.92–2.17) ^a^	2.30 (2.08–2.52) ^b^
**20–39 years**				
Mean (95% CI)	1.98 (1.83–2.13) ^a^	1.76 (1.44–2.07)	1.95 (1.80–2.10)	2.19 (1.93–2.44)
**40–59 years**				
Mean (95% CI)	2.04 (1.87–2.21) ^a,b^	1.89 (1.68–2.10)	1.99 (1.81–2.17)	2.28 (2.01–2.55)
**≥60 years**				
Mean (95% CI)	2.28 (2.15–2.41) ^b^	1.96 (1.67–2.24)	2.25 (2.11–2.40)	2.60 (2.25–2.95)

EPA: eicosapentaenoic acid; DPA: docosapentaenoic acid; DHA: docosahexaenoic acid. Data shown are weighted means and 95% confidence intervals. Comparisons were tested for age groups within the all column and for race groups within age rows. Means with different subscripts vary significantly, *p* < 0.00325.

**Table 3:** Prevalence of inadequate LCn-3 levels for cardiovascular health.

*N* (%)	All	Hispanic	Non-Hispanic White	Non-Hispanic Black
Below dietary recommendations; LCn-3 plasma PUFA < 2.49%				
≥20 years	1109 (80.6)	311 (91.2) ^a^	588 (82.0) ^a,b^	178 (73.2) ^b^
20–39 years	352 (84.2)	98 (94.7)	165 (83.6)	78 (78.7)
40–59 years	351 (81.8)	90 (87.5)	190 (84.6)	58 (76.1)
≥60 years	406 (72.9)	123 (85.2)	233 (75.5)	42 (53.6)
Below concentrations associated with cardio-protection ^b^; LCn-3 plasma PUFA < 3.86%				
≥20 years	1322 (95.7)	328 (98.2)	707 (96.0)	235 (93.8)
20–39 years	399 (96.9)	100 (98.2)	191 (97.5)	94 (94.5)
40–59 years	401 (95.0)	94 (98.2)	214 (95.8)	71 (92.4)
≥60 years	522 (94.8)	134 (97.9)	302 (94.5)	70 (94.3)

LCn-3 PUFA: long-chain omega-3 polyunsaturated fatty acid. ^a^ The LCn-3 concentration approximately equivalent to dietary intake of 2.60 mg/day of EPA and DHA from Sun *et al.* [9]; ^b^ the lower range of LCn-3 the second tertile from Virtanen *et al.* [17] associated with 53% lower risk of sudden cardiac death. Means with different subscripts vary significantly, *p* < 0.00325.

**Figure 1:**
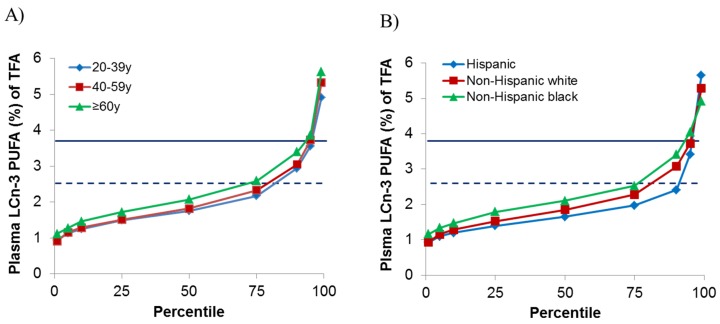
Serum long chain *n*-3 fatty acids by age (**A**) and race/ethnicity (**B**) in NHANES 2003–2004. Sum of eicosapentaenoic acid, docosahexaenoic acid and docosapentaenoic acid/total fatty acids. The solid line represents LCn-3 PUFA of 3.86%, the lower range of LCn-3 in the second tertile from Virtanen *et al.* [17] that was associated with 53% lower risk of sudden cardiac death. The dashed line represents LCn-3 PUFA of 2.49% a concentration approximately equal to dietary recommendations [1,5,7].

Figure 1 shows the mean distribution of plasma LCn-3 PUFA concentrations in percentiles by age groups (A) and race (B). Non-Hispanic white and non-Hispanic black participants of all ages have LCn-3 PUFA below concentrations associated with dietary recommendations until approximately the 75th percentile while Hispanic participants meet the threshold until beyond the 90th percentile. Concentrations are below those associated with cardio-protective benefits until approximately the 95th percentile for all races and ages.

## 4. Discussion

This study provides a descriptive overview of plasma LCn-3 PUFA concentrations in a nationally representative population of American adults. The mean LCn-3 PUFA concentrations of only 2% in men and women likely reflects the low consumption of fish and seafood that predominates in the U.S. and many other industrialized nations [6]. Approximately 8 in 10 participants had LCn-3 PUFA concentrations below a level associated with meeting the dietary recommendations to consume two servings of fatty fish like salmon, mackerel, herring, lake trout, sardines and albacore tuna per week. This is of importance given the role of LCn-3 PUFA for overall health [19] as well as cardiovascular health [20]. More than 9 in 10 had LCn-3 PUFA concentrations below a level associated with reduced risk of cardiac death [17]. Estimates project that by 2030, 40.5% of the U.S. population will have some form of CVD and the real indirect costs for all CVD are estimated to increase by 61% from 2010 to 2030 [21]. A systematic review estimated that increasing omega-3 intake and status could result in a 6.9% reduction in the incidence of CVD-related events and avoid hospital utilization by $2.06 billion per year among U.S. adults over 55 years, suggesting that improving LCn-3 PUFA intake, and by extension status, could have significant cost implications for the U.S. healthcare system [22].

The data presented here using a criterion of adequacy for an LCn-3 PUFA index relative to cardiovascular health will be useful for providing a frame of reference to help interpret the results of previous studies as well as future studies on omega-3 fatty acids. For example, associations between LCn-3 PUFA and health outcomes could be weighed against where the study participants’ LCn-3 PUFA status is relative to the distribution in NHANES to determine if status is optimal or suboptimal. LCn-3 PUFA is particularly likely to be suboptimal for Hispanic participants and participants younger than 60 years. The finding of differences in LCn-3 PUFA by age and race may help focus efforts to increase LCn-3 intakes especially given the growing Hispanic population within the U.S. [23] and the large proportion of the population aged 20–59 years.

### Strengths and Limitations

A strength of this study is the measure of LCn-3 PUFA’s in plasma rather than from estimates of dietary intake, which are prone to misreporting. A further strength is the large sample size that is representative of the U.S. population. The eligible sample for fatty acids was drawn from participants aged 20 and older in NHANES. Similar data from children and adolescents would be valuable, especially in light of the important role of LCn-3’s in cognitive behavior/maturation [24] and childhood developmental disorders [25,26]. A limitation of this study is that plasma LCn-3 PUFA concentrations represent both shorter term dietary consumption as well as endogenous synthesis of fatty acids, and therefore may not necessarily be indicative of long-term dietary intake. Red blood cells would provide a better indication of longer term intake of LCn-3 PUFA [9], however data regarding red blood cell LCn-3 PUFA status was not collected in NHANES.

The timeliness of the data also warrants consideration. Dietary intake of fish and seafood has remained consistently low in the U.S. over the past two decades [27] but fish oil supplement use has increased among adults [28] raising the possibility that contemporary LCn-3 PUFA concentrations are higher than these data show. However, in 2012 only 7.8% of adults reported fish oil use [28] which is likely too low to have a population level impact on LCn-3 PUFA status. We did not stratify plasma LCn-3 PUFA by the dietary source (fortified, naturally occurring or supplements) as there were only 45 participants who reported use of supplements containing EPA, DHA or DPA. With the growing prevalence of fortified foods and supplements it may be possible to examine the source of LCn-3 PUFA in future analyses.

## 5. Conclusions

Suboptimal LCn-3 PUFA concentrations are common among U.S. adults despite repeated emphasis on increasing seafood consumption. Hispanic individuals and individuals aged 20 to 59 may be particularly important populations to focus on given the high prevalence of suboptimal LCn-3 PUFA. Notably, no subpopulation had a majority of individuals with LCn-3 concentrations above suboptimal, thus highlighting the need for improving LCn-3 PUFA levels in the food supply.

## Supplementary Materials

The following are available online at http://www.mdpi.com/2072-6643/7/12/5534/s1, Table S1: Fatty acid status (% of total fatty acids) measured in adults aged 20 and older in NHANES 2003–2004, Table S2. Long chain *n*-3 PUFA status (% of total fatty acids: EPA + DPA + DHA) in select percentiles in all participants and by race and age.
