# Supplementary material for: Suboptimal Plasma Long Chain n-3 Concentrations are Common among Adults in the United States, NHANES 2003–2004
Source: Nutrients. 2015 Dec 9;7(12):10282–9. doi: 10.3390/nu7125534 (PMC4690086; doi:10.3390/nu7125534)
Supplement: Supplementary file 1 [file nutrients-07-05534-s001.docx]

Supplementary Materials: Suboptimal Plasma Long Chain *n*-3 Concentrations are Common among Adults in the United States, NHANES 2003–2004

Rachel A. Murphy, Elaine A. Yu, Eric D. Ciappio, Saurabh Mehta and Michael I. McBurney

**Table S1.** Fatty acid status (% of total fatty acids) measured in adults aged 20 and older in NHANES 2003–2004.

| **Fatty Acid** | **All Mean (95% CI)** |
| --- | --- |
| Myristic acid | 1.14 (1.10–1.18) |
| Myristoleic acid | 0.07 (0.07–0.08) |
| Palmitic acid | 24.3 (24.0–24.6) |
| Palmitoleic acid | 2.12 (2.04–2.20) |
| Stearic acid | 6.27 (6.17–6.37) |
| Oleic acid | 19.0 (18.8–19.3) |
| *cis*-Vaccenic acid | 1.32 (1.28–1.36) |
| Linoleic acid | 31.2 (30.8–31.7) |
| γ-Linolenic acid | 0.46 (0.44–0.47) |
| α-Linolenic acid | 0.59 (0.58–0.61) |
| Homo-γ-Linolenic acid | 1.38 (1.36–1.40) |
| Arachidic acid | 0.22 (0.21–0.22) |
| Eicosenoic acid | 0.13 (0.12–0.13) |
| Eicosadienoic acid | 0.19 (0.19–0.20) |
| Arachidonic acid | 7.17 (7.07–7.27) |
| Docosanoic acid | 0.65 (0.62–0.67) |
| Docosenoic acid | 0.04 (0.04–0.05) |
| Docosatetraenoic acid | 0.23 (0.23–0.23) |
| Docosapentaenoic-6-acid | 0.18 (0.18–0.19) |
| Lignoceric acid | 0.50 (0.49–0.52) |
| Nervonic acid | 0.71 (0.68–0.73) |

Data shown are weighted means and 95% confidence intervals. Fatty acids shown represent 21 of the 24 fatty acids measured in NHANES. Together with eicosapentaenoic acid, docosapentaenoic-3 acid and docosahexaenoic acid (shown in the manuscript tables) were summed to generate total fatty acids to derive the relative concentration (%) of fatty acids.

**Table S2.** Long chain *n*-3 PUFA status (% of total fatty acids: EPA + DPA + DHA) in select percentiles in all participants and by race and age.

| **Percentile** | **All** | **Hispanic** | **Non-Hispanic White** | | **Non-Hispanic Black** |
| --- | --- | --- | --- | --- | --- |
| **≥20 years** | | | | | |
| 10th (95% CI) | 1.14 (1.12–1.17) | 1.09 (1.05–1.13) | 1.14 (1.10–1.17) | | 1.32 (1.27–1.37) |
| 50th (95% CI) | 1.79 (1.78–1.79) | 1.60 (1.59–1.62) | 1.77 (1.76–1.78) | | 2.06 (2.04–2.08) |
| 90th (95% CI) | 2.76 (2.71–2.80) | 2.23 (2.19–2.27) | 2.69 (2.64–2.74) | | 3.02 (2.96–3.07) |
| **20–39 years** | | | | | |
| 10th (95% CI) | 1.11 (1.06–1.16) | 1.07 (1.00–1.13) | 1.10 (1.05–1.15) | | 1.29 (1.21–1.37) |
| 50th (95% CI) | 1.71 (1.70–1.72) | 1.57 (1.55–1.59) | 1.71 (1.69–1.72) | | 1.99 (1.96–2.01) |
| 90th (95% CI) | 2.54 (2.47–2.60) | 2.09 (2.05–2.12) | 2.57 (2.47–2.66) | | 2.86 (2.63–3.09) |
| **40–59 years** | | | | | |
| 10th (95% CI) | 1.13 (1.08–1.18) | 1.12 (1.11–1.13) | 1.13 (1.06–1.20) | 1.34 (1.24–1.44) | |
| 50th (95% CI) | 1.73 (1.71–1.75) | 1.60 (1.59–1.62) | 1.77 (1.76–1.79) | 2.06 (2.03–2.08) | |
| 90th (95% CI) | 2.71 (2.65–2.77) | 2.23 (2.17–2.29) | 2.69 (2.59–2.81) | 2.99 (2.87–3.10) | |
| **≥60 years** | | | | | |
| 10th (95% CI) | 1.27 (1.23–1.31) | 1.11 (1.02–1.19) | 1.29 (1.19–1.38) | 1.54 (1.35–1.73) | |
| 50th (95% CI) | 1.99 (1.97–2.01) | 1.66 (1.61–1.70) | 1.94 (1.93–1.95) | 2.32 (2.28–2.35) | |
| 90th (95% CI) | 3.13 (3.06–3.20) | 2.23 (2.18–2.28) | 2.86 (2.74–2.99) | 3.91 (3.51–4.32) | |

Data shown are weighted means and 95% confidence intervals.
